# The experience of demoralization syndrome in patients with decompensated cirrhosis: A qualitative research

**DOI:** 10.1371/journal.pone.0337182

**Published:** 2025-12-01

**Authors:** Xin Shu, Ruxiu Xiong, Yubiao He, Xue Li, Guifei Li, Cailiang Qiu

**Affiliations:** 1 Department of Infectious Diseases, The Second Xiangya Hospital of Central South University, Changsha, Hunan, China; 2 Department of Nursing, Hunan Normal University School of Medicine, Changsha, Hunan, China; Government Villupuram Medical College and Hospital, INDIA

## Abstract

Demoralization syndrome presents a significant psychological challenge for patients grappling with decompensated cirrhosis, detrimentally impacting their prognosis and quality of life. This study aims to elucidate the experience of demoralization syndrome in these patients and to inform the development of targeted interventions. Through purposeful sampling, semi-structured interviews were carried out with patients receiving treatment at tertiary hospitals in Changsha City, Hunan Province, China, from July to November 2023. Data analysis employed Colaizzi’s seven-step method. Among the 18 participants—comprising 12 males and 6 females, aged between 27 and 60 years—interview data revealed 4 themes and 11 sub-themes, including sources of demoralization syndrome (internal and external factors); impact of demoralization syndrome (sleep disturbance, suicidal ideation, social isolation); coping strategies of demoralization syndrome (submission and endurance, avoidance and concealment, acceptance of confronting); mitigation of demoralization syndrome support needs (medical resources, emotional catharsis, social support). Given the diverse sources and implications of demoralization syndrome in patients contending with decompensated cirrhosis, healthcare professionals should prioritize psychological assessment and formulate multidimensional intervention strategies to alleviate its severity.

## Introduction

Decompensated cirrhosis, also known as end-stage liver disease, is characterized by symptoms of portal hypertension and hepatic decompensation, as well as complications such as ascites, gastrointestinal bleeding, sepsis, hepatic encephalopathy, hepatorenal syndrome, and carcinoma often occur [1]. Globally, there are 120 million patients with cirrhosis, of which 10.6 million are in the decompensated phase, and about 5% to 7% progress to decompensated phase each year [2]. Currently, drug therapy serves as the primary treatment, but its effectiveness in preventing and controlling complications is limited. Liver transplantation is the only effective treatment option for end-stage liver disease; however, it is constrained by issues such as liver source availability and cost. Consequently, the prognosis of the disease is generally poor, and in the case of advanced loss of compensation, the case fatality rate reaches as high as 60% ~ 80% within one year, which seriously impairs the quality of life and reduces the life expectancy of patients [1,3]. Moreover, patients with decompensated cirrhosis face external pressure from various aspects such as economy and society, and often suffer from psychological pains like despair, frustration and sadness. These factors hinder their ability to devote themselves to treatment, and may even lead to thoughts of abandoning treatment [4]. Demoralization syndrome, First proposed in 2002 by Australian scholars Clarke et al [5], refers to a patient’s lack of psychological regulation or adaptive strategies in the face of chronic stressors, resulting in persistent feelings of helplessness, hopelessness, meaninglessness, coping incompetence, and self-esteem deficits. Studies have shown that 27.4% to 59.1% of patients with chronic illnesses have demoralization syndromes [6]. The majority of previous research on demoralization syndrome has focused on patients with cancer [7], maintenance hemodialysis [8], and chronic heart failure [9]; studies on patients with decompensated cirrhosis are scarce, and most of them have been cross-sectional in nature, with limited attention given to the patients’ emotional experience and underlying causes. Qualitative studies, in particular, are lacking in the investigation of patients with decompensated cirrhosis. Therefore, this study adopted a qualitative design using descriptive phenomenology and semi-structured interviews with 18 patients with decompensated cirrhosis to explore their experiences and feelings related to demoralization syndrome. The findings from this study will contribute to the development of targeted intervention strategies.

## Materials and methods

### Study design

This study adopted a qualitative descriptive phenomenological design and followed the Consolidated Criteria for Reporting Qualitative Research (COREQ) guidelines [10].

### Participants

A cross-sectional study was conducted from July to November 2023 at a tertiary-level hospital in Changsha City, Hunan Province, China, to investigate the demoralization syndrome among patients with decompensated cirrhosis. The demoralization scale-mandarin version (DS-MV) (S1 File), which was translated into Mandarin by Hong Xiaoqi et al, was used for assessment, and those with DS-MV scores ≥30 were defined as clinically significant [11]. The inclusion criteria were: adults (≥18 years) with decompensated cirrhosis; no clinical diagnosis of hepatic encephalopathy (screened using the West Haven Hepatic Encephalopathy Grading Scale [WHGES], grade 0); DS-MV score ≥30; voluntary participation in audio-recorded interviews; and written informed consent. The exclusion criteria were: overt hepatic encephalopathy (screened using the WHGES, grade ≥II) or other severe cognitive impairment (assessed by the Mini-Mental State Examination [MMSE], score <24, or the Confusion Assessment Method [CAM], positive for delirium); comorbid severe psychiatric disorders (diagnosed according to DSM-5 criteria by a psychiatric specialist); impaired verbal communication; or medical instability or terminal illness. All screenings were conducted by the study’s attending physician within 24 hours prior to enrollment. In our previous quantitative study, a total of 444 patients with decompensated cirrhosis were included. For the qualitative interviews, 318 patients with a DS-MV score of 30 or above were screened as potential candidates. From this group, participants were selected using the purposeful sampling method to ensure maximum variation, minimize selection bias, and capture diverse perspectives. The final sample size was determined based on data saturation, defined as the point at which no new themes or concepts emerged across three consecutive interviews. Ultimately, 18 participants were included, comprising 6 females and 12 males, aged 27–60 years. Basic information about the interviewees is shown in Table 1. Prior to commencement, the study received ethical approval from the Biomedical Research Ethics Committee of Hunan Normal University, China (Approval No. 2023337). Informed consent forms were signed by all participants prior to their inclusion in the study.

**Table 1:** Socio-demographic and clinical characteristics of the study participants.

No.	Sex	Age (years)	Education	Monthly income (CNY)	Etiology	Duration time (years)
P1	Female	49	Primary school or less	<1000	Post-infectious hepatitis	<1
P2	Male	60	Primary school or less	<1000	Alcoholic cirrhosis	6 ~ 10
P3	Male	53	Middle school	<1000	Post-infectious hepatitis	<1
P4	Male	39	Middle school	2000 ~ 3000	Post-infectious hepatitis	1 ~ 5
P5	Female	44	Middle school	2000 ~ 3000	Primary biliary cirrhosis	6 ~ 10
P6	Male	42	Middle school	>5000	Post-infectious hepatitis	<1
P7	Male	49	Middle school	<1000	Post-infectious hepatitis	>10
P8	Female	41	Middle school	3000 ~ 4000	Post-infectious hepatitis	6 ~ 10
P9	Male	31	High school or secondary school	>5000	Post-infectious hepatitis	1 ~ 5
P10	Female	33	Middle school	<1000	Others	1 ~ 5
P11	Male	27	College degree or above	>5000	Others	6 ~ 10
P12	Female	33	College degree or above	1000 ~ 2000	Others	>10
P13	Male	31	High school or secondary school	>5000	Alcoholic cirrhosis	1 ~ 5
P14	Male	37	College degree or above	2000 ~ 3000	Others	1 ~ 5
P15	Male	53	Middle school	2000 ~ 3000	Alcoholic cirrhosis	<1
P16	Female	54	Middle school	<1000	Post-infectious hepatitis	1 ~ 5
P17	Male	43	College degree or above	>5000	Others	1 ~ 5
P18	Male	44	Middle school	<1000	Others	6 ~ 10

### Instrument

Based on the findings of our previous quantitative study, a systematic literature review was conducted using the keywords “decompensated cirrhosis,” “qualitative study,” and “demoralization syndrome.” From this review, three core interview dimensions were identified: “disease-related stressors,” “coping strategies,” and “social support,” with the Transactional Model of Stress and Coping adopted as the overarching theoretical framework [12]. Guided by these dimensions and the framework, we initially designed five open-ended questions. The draft interview guide was reviewed by two qualitative research methodology experts and a chief physician in infectious diseases to assess its content validity, clinical relevance, and linguistic clarity. The researchers then conducted pilot interviews with two eligible patients, followed by preliminary analysis of the transcripts. Based on expert feedback and practical experience, the interview guide was revised to produce the final framework used for the formal study (S2 File).

### Data collection

All interviews were conducted by the first author, a graduate student and female nurse with systematic training in psychological interviewing methods and techniques, extensive qualitative research experience, and strong proficiency in applying interview skills. Prior to the interviews, tranquil venues within the infectious disease department were carefully selected, and interview times were scheduled in consultation with the participants. During the interviews, access to the room was restricted to protect participants’ privacy. Immediately before the interview, a brief cognitive screen was conducted to ensure participants’ cognitive soundness, including a 60-second Animal Naming Test and orientation/attention checks, and observation for clinical signs of hepatic encephalopathy (e.g., asterixis or fluctuating attention). If cognitive impairment was detected at this point or during the interview, the session was discontinued and rescheduled after clinical stabilization. There was no prior contact between the researchers and the participants before the study. Prior to each interview, the purpose and procedures of the study were explained to each participant, and written informed consent was obtained. A semi-structured interview guide, designed for open-ended questioning, was employed to systematically gather data. Interviews were conducted in Chinese, as it is the participants’ native language. Interviews were conducted in real-time, typically spanning 20–30 minutes, and audio-recorded using an iFLYTEK Smart Recorder (model B1) for accuracy. In addition to audio-recorded verbal responses, the first author took field notes during each interview to document participants’ non-verbal behaviors and emotional expressions, such as crying, laughing, signs of anger, or anxiety. These field notes were used alongside the transcripts during data analysis to ensure a comprehensive understanding of participants’ experiences. During the interviews, the interviewer consistently adhered to the principles of neutrality, non-judgment, and non-criticism, avoiding any guidance or suggestion. At the same time, techniques such as attentive listening, clarification, probing, and repetition were employed, with flexible adjustments to the interview questions to ensure that participants could freely and fully express their views. Interviews concluded when saturation was reached, signifying no new insights emerged. Each interview was transcribed within 24 hours.

### Data analysis

Qualitative data were analyzed using NVivo 12.0 software and interpreted following Colaizzi’s seven-step method [13]. To ensure anonymity, all identifiers were removed and replaced with unique codes (P1–P18) by the first author. The first author transcribed all interviews verbatim in Chinese by listening to the audio recordings, and the second author verified the transcripts for accuracy; any unclear or ambiguous segments were replayed multiple times and jointly reviewed, with uncertain passages annotated (e.g., “inaudible/unclear”) and resolved through discussion, taking the audio as authoritative. Both authors independently and repeatedly reviewed the transcripts to gain a deep understanding of the data and to identify significant statements related to the experience of demoralization syndrome. Meaningful data segments were extracted and assigned initial codes (e.g., “fear,” “despair,” “failure”), which were systematically compared, color-coded, and grouped into categories based on thematic similarities and differences. Through an iterative process, overarching themes were identified and comprehensively described. To ensure coding consistency, the first and second authors conducted independent analyses and compared their results, discussing any discrepancies with all researchers until consensus was reached.

### Methodological rigor

We ensured trustworthiness by following established qualitative criteria of credibility, dependability, confirmability, and transferability [14]. All interviews were transcribed verbatim in Chinese by the first author and independently verified by the second; ambiguities were resolved through repeated playback and discussion. Dependability was maintained using NVivo 12.0 and Colaizzi’s seven-step method, with coding histories and decision logs preserved. Both authors coded independently and reconciled discrepancies through consensus. Confirmability was supported by an audit trail, analytic memos, and reflexive team discussions, with themes grounded in verbatim quotations. Transferability was addressed by providing detailed descriptions of the study setting, participant characteristics (P1–P18), and illustrative quotes, while de-identification ensured confidentiality. The completed COREQ checklist is provided in the Supporting Information (S3 File).

## Results

### Sources of demoralization syndrome

#### Internal factors.

a Somatic symptoms

Decompensated cirrhosis is a severe stage of liver disease characterized by multiple systemic disturbances, including malnutrition, altered metabolic status, inflammation, and endocrine dysfunction. These abnormalities contribute to the progressive deterioration of liver function, often accompanied by symptoms such as fatigue, loss of appetite, pain in the hepatic region, and cognitive impairment.

*“During the onset…um…my weight just…noticeably went down. My belly…it was…uh…swollen with ascites, so it was hard to eat. I felt full even when I didn’t eat much, and just thinking about food…made me feel nauseous. I could only…like…drink a little water each day.” (Participant P5, female, 44years)*

*“I… don’t have the strength. I feel like… an invalid. I… can’t do anything.” (Participant P10, female, 33 years)*

b Uncertainty of disease

Many patients grapple with the unpredictable nature of their illness, compounded by limited access to disease-related information and difficulties in comprehending and accepting their condition. This lack of understanding engenders feelings of powerlessness and a diminished sense of control over their health and future.

*“When I feel down, I skip my medications; when my symptoms get worse, I’m even less willing to stick with treatment.” (Participant P16, female, 54 years)*

c Poor prognosis of disease

Patients with advanced decompensated cirrhosis often face a protracted and challenging treatment course with a bleak prognosis. Prolonged treatment durations can lead to skepticism regarding treatment efficacy and a gradual erosion of confidence in recovery.

*“I have undergone artificial liver treatment several times, but instead of improving, my jaundice worsened.” (Participant P1, female, 49 years)*

*“I’ve only been out of the hospital for less than a week, and I’m back in the hospital again, over and over again, and I don’t know when it’s going to end.” (Participant P18, male, 44 years)*

#### External factors.

a Financial pressure

Most of the patients have to bear heavy medical expenses due to the high costs associated with treating their illnesses, and their financial burden is further aggravated by the decline or loss of their labor force, which deprives their families of their main source of income.

*“I can’t work as I used to, but the bills keep piling up. I need over 2,000 yuan a month after reimbursement for medications, and I have to spend another 10,000 to 20,000 yuan in cash for each hospitalization, which works out to at least 70,000 to 80,000 yuan a year.” (Participant P18, male, 44 years)*

b Fear of being a burden to family

The shift in the patient’s family role has led to a decline in their family functioning, and the fear of dragging their family members down has led to an overburdening of their psyche and aggravated demoralization syndrome.

*“With my wife caring for our two children at home and my father having to come to the hospital to take care of me, I feel like I’m burdening my family.” (Participant P9, male, 31 years)*

*“My father-in-law, who is diabetic himself, came to the hospital for a review this time, and now he has to come and take care of me.” (Participant P17, male, 43 years)*

c Restriction of social life

Prolonged and uninterrupted treatment destroys patients’ daily routines and social interactions, resulting in a loss of their normal social lives.

*“I’ll undoubtedly need an extended leave from work upon my return. Teaching classes this semester is out of the question, and I’ll need to delegate my responsibilities to colleagues. While this will undoubtedly pose challenges, I have no other option.” (Participant P17, male, 43 years)*

d Social stereotypes

Many patients perceive societal prejudices against them, leading to increased anxiety and depression.

*“People in my husband’s village speculate that my illness at such a young age must be a result of familial misfortune or past transgressions. They even suggest that my husband’s misfortune in marrying me is to blame.” (Participant P10, female, 33 years)*

*“There’s a prevailing belief that my illness is contagious, which fills me with fear of inadvertently transmitting it to my grandchildren.” (Participant P15, male, 53 years)*

### Impact of demoralization syndrome

#### Sleep disturbance.

Some patients with decompensated cirrhosis reported that they experienced disrupted sleep patterns, attributable to physical and mental tension induced by the progression of the disease, environmental changes, and heightened pre-sleep anxiety.

*“I have consistently struggled with sleep, and amidst the challenges at work and home over the past few years, I have resorted to alcohol as a means to numb myself so as to attain sleep. Now, with the flare-up and increased contemplation, my ability to sleep has significantly diminished.” (Participant P13, male, 31 years)*

#### Suicidal ideation.

Faced with the psychological stress brought on by the disease, some patients experience a sense of despair and engage in self-harming behaviors, including suicidal thoughts.

*“I frequently find solace in the thought of death. While I came to the hospital seeking treatment this time, I have also contemplated seeking euthanasia.” (Participant P12, female, 33 years)*

#### Social isolation.

Most patients reported a diminished sense of self-worth due to changes in their appearance, impaired work efficiency, and fear of aggravating their condition by socializing, leading to withdrawal from social activities.

*“Well, in fact, at our age, um, friends are few and far between, especially when you are sick, and, you know, if you go back to communicating with others, they seem to think you have something else on your mind…” (Participant P17, male, 43 years)*

### Coping strategies of demoralization syndrome

#### Submission and endurance.

Most patients reported difficulty in taking effective control measures in the face of physical and mental distress, and they had no choice but to cope by giving in and enduring.

*“I was, um, born to have this disease… what can be done?” (Participant P7, male, 49 years)*

#### Avoidance and concealment.

Some patients instinctively hid or denied the existence and severity of the disease out of a kind of self-selection to alleviate their psychological suffering.

*“I seldom discuss my illness openly. Instead, I internalize it, striving to overcome it alone.” (Participant P6, male, 42 years)*

*“Overthinking only leads to disappointment, so it’s better not to think about it.” (Participant P16, female, 54 years)*

#### Acceptance of confronting.

Some patients experienced post-traumatic growth, gaining deeper insights into the value and purpose of life. They actively adopted coping strategies such as diversion of attention and positive psychological suggestion to manage negative emotions, alleviate or eliminate bad habits in their lives, establish a healthy lifestyle, and mitigate the impact of the disease.

*“Well, following this ordeal… I have, um, resolved to abstain from alcohol consumption.” (Participant P13, male, 31 years)*

### Mitigation of demoralization syndrome support needs

#### Medical resources.

Some patients expressed dissatisfaction with primary healthcare facilities, citing a lack of specialized professionals or perceived deficiencies in cognitive and technical capabilities. They tended to go to large hospitals with specialized services, strong technical competence and a high level of satisfaction for consultation.

*“Given the limitations of local medical services, I have to resort to seeking treatment at larger hospitals.” (Participant P4, male, 39 years)*

*“Mental health concerns are often overlooked in our locality.” (Participant P14, male, 37 years)*

#### Emotional catharsis.

Some of the patients lamented the inadequacy of emotional support from their families, noting a deficiency in meaningful spiritual and emotional exchanges despite receiving material assistance.

*“My marriage… lacks emotional depth; futile attempts to communicate with my husband… yield little solace.” (Participant P8, female, 41 years)*

*“I am unmarried and live with my mom. My siblings sometimes give some financial support but we rarely talk.” (Participant P16, female, 54 years)*

#### Social support.

Patients emphasized the importance of robust social support systems, including improved healthcare insurance coverage, enhanced drug reimbursement policies, and increased familial encouragement and assistance, which significantly bolster their will to persevere.

*“I… wanted to give up several times, but all those cousins gave me money and called to tell me to stick with the treatment.” (Participant P6, male, 42 years)*

## Discussion

### Diverse sources of demoralization syndrome in patients with decompensated cirrhosis

Our findings indicate that disease‑intrinsic factors (e.g., somatic symptoms, illness uncertainty) together with disease‑external factors (e.g., financial pressure, social stereotypes) constitute the two principal triggers of demoralization syndrome among patients with decompensated cirrhosis. Participants frequently reported frustration and despair stemming from recurrent complications and complex treatment regimens (“I feel like an invalid. I can’t do anything”). Many also described limited access to disease-related knowledge following a late-stage diagnosis. Confronted with uncertainty about disease progression, they struggled to understand the illness and cope effectively, which engendered loss of control, helplessness, and a gradual erosion of future confidence. This uncertainty, together with low self-efficacy, perpetuates a vicious cycle in which low mood undermines adherence, leading to symptom worsening and, ultimately, greater despair (“When I feel down, I skip my medications; when my symptoms get worse, I’m even less willing to stick with treatment”). Demographically, our sample reflected a younger age at onset, with most participants serving as their family’s primary earners. Reduced work capacity, together with high medical costs, increased psychological stress by imposing the dual burden of diminished earnings and greater financial obligations (“I can’t work as I used to, but the bills keep piling up”). Aligned with these findings, Handoyo et al. [15] reported that for men who measure self-worth by their role in “providing for the family,” illness can jeopardize their identity and bodily integrity. Participants in our study expressed analogous threats, articulated as guilt, shame, and anxiety, emotions they often considered more distressing than the physical symptoms of decompensated cirrhosis. In our interviews, some participants reported feeling judged when seeking medical care, particularly when their condition was attributed to alcohol abuse and high-risk sexual behaviors, making them more likely to experience pronounced societal stigmatization. This underscores societal stigmatization as a deep-seated trigger of demoralization syndrome and resonates with the qualitative findings of Toumi et al., which documented a pervasive ‘deserved illness’ stereotype among patients with liver disease regardless of etiology [16]. Our data ground this conclusion with concrete examples and further show how such stigmatization exacerbates self-loathing and fosters a pervasive sense of hopelessness. Taken together, these insights highlight the need for destigmatizing clinical communication, routine assessment of demoralization syndrome, and timely psychosocial referral in hepatology care.

### Focusing on positive patient emotions to guide positive coping

Coping styles represent the cognitive and behavioral strategies individuals employ when confronting stress and frustration [17]. Our study delves into how patients with decompensated cirrhosis navigate these coping mechanisms, highlighting the presence of two distinct approaches: negative coping and positive coping. This finding is consistent with previous research conducted by Fang Fang et al. [18]. In the narratives of negative coping, patients often exhibit a tendency to succumb to the persistent distress caused by their illness. Our in-depth interviews further reveal that this coping mechanism is not only rooted in the reality of the uncontrollable nature of complications and the pressures of poor prognosis but also deeply ingrained in patients’ feelings of helplessness regarding their disease progression. Many participants disclosed psychological motivations behind “giving up” or “avoiding discussions about their condition.” One participant candidly expressed, “Overthinking only leads to disappointment, so it’s better not to think about it.” This perspective not only reflects an emotional avoidance on the individual level but also reveals the subjective uncertainty they experience when facing their illness. This uncertainty often triggers fear and anxiety, prompting patients to conceal their health issues, thereby falling into a vicious cycle of psychological withdrawal. When patients choose not to disclose their health issues, they are unable to access crucial support, leading to increased feelings of helplessness and isolation that ultimately diminish their confidence and willingness to confront the illness. Additionally, social stigma significantly affects patients’ coping strategies. Some participants clearly stated that, fearing the label of “liver disease,” they choose to hide their conditions, refuse social support, and even avoid follow-up visits. Conversely, positive coping is characterized by patients’ proactive acceptance and reconstruction of their illness experience, typically manifested through the recognition of post-traumatic growth, resilience in facing their realities, and a gradual exploration and reconstruction of the meaning of life through ongoing reflection on their illness. This study found that patients with chronic liver disease who employed such coping strategies generally exhibited higher levels of life satisfaction and emotional well-being, a finding that aligns with the results of Stewart et al. [19], further supporting the important role of positive coping in reducing feelings of hopelessness and promoting the development of psychological adaptation among patients. In light of these findings, it is imperative for clinical nurses to integrate a holistic understanding of the psychological landscape of patients with decompensated cirrhosis into their caregiving approach. By emphasizing positive psychology interventions, nurses can empower patients to embrace self-acceptance, share moments of joy, and cultivate optimistic outlooks. These interventions serve to augment patients’ psychological elasticity, fortify their resilience against stressors, and foster a proactive stance towards managing their condition. Ultimately, such initiatives aim to mitigate the onset of demoralization syndrome among this patient population.

### Providing avenues for emotional catharsis and facilitating patient role change

Studies have shown that patients with decompensated cirrhosis often seek emotional catharsis primarily from their spouses, parents and friends [20]. In-depth interviews conducted for this study further revealed that most patients with decompensated cirrhosis have substantial emotional expression needs and a desire for support. Particularly when facing significant psychological stress, patients strongly hope that their family members can provide not only emotional acceptance and companionship but also substantial professional assistance in medical decision-making and daily management. However, many participants noted that due to a lack of safe, empathetic, and effective communication channels, they often feel compelled to conceal their true emotions and psychological struggles. Effective emotional catharsis plays a pivotal role in bolstering patients’ psychological resilience and self-regulatory capabilities, enabling them to navigate the adversities accompanying their condition and fostering a more positive outlook [21]. Recognizing the pivotal role of family members in detecting psychological shifts in patients [20], nursing staff should help family members understand the helplessness and hopelessness of the patient. This entails guiding them in responding empathetically to the patient’s emotional outbursts, fostering open communication channels, and striking a delicate balance between concern and overbearing attention. Importantly, healthcare providers should be enlisted whenever necessary to provide specialized assistance. In addition, it was also found that patients with decompensated cirrhosis lacked skills in emotional communication and tended to choose the coping strategy of concealment, which would weaken the patients’ self-regulation ability, reduce their sense of well-being and social belonging, and might even cause them to miss the optimal time to receive psychological interventions [17]. Therefore, healthcare professionals are encouraged to facilitate role changes among patients, steering them away from passive concealment towards active engagement with their emotions. Innovative approaches such as board games and problem-based teaching methods can serve as effective conduits for patients to unburden themselves emotionally and rediscover the purpose and meaning in their lives through interactions with trusted individuals and professionals.

### Improving the social support system to meet the multidimensional needs of patients with decompensated cirrhosis

This study identified the multifaceted needs of patients with decompensated cirrhosis in coping with demoralization syndrome. Prolonged illness and financial strain often precipitate psychological shifts and negative emotions – which profoundly impact patients’ physical and mental well-being, as well as their adherence to healthy behaviors [22]. Following the onset of the disease, patients with decompensated cirrhosis experience a shift in their role from caregiver to cared-for, with a significant drop in their total household income and rising medical expenses. This exacerbates their family’s financial burden, necessitating increased social support to mitigate the stress of illness. Therefore, patients should assess their family’s financial status and collaborate with healthcare professionals to devise cost-effective diagnosis and treatment plans, minimizing unnecessary medical expenditures. Timely application for governmental subsidies and participation in charitable initiatives such as Water Drop Fundraising can provide crucial financial aid from governmental agencies, organizations and compassionate individuals. This interview revealed patients’ concerns regarding the lack of accessible professional guidance beyond hospital settings. Disparities in regional development and limited healthcare access in remote areas, particularly rural regions, pose significant barriers to fulfilling recommended medical practices despite patients’ awareness of the importance of disease screening [23]. Therefore, government departments should speed up and optimize the establishment of a hierarchical diagnosis and treatment system to alleviate the imbalance of medical resources and improve the efficiency of their utilization. Medical institutions should prioritize the formation of comprehensive nursing team and service frameworks to deliver ongoing post-discharge care and address patients’ knowledge gaps, facilitating their recovery and condition stabilization. Furthermore, psychosocial support emerged as a critical factor in patients’ adaptation to the illness. Under the prolonged stress of living with the disease, participants relied on networks of healthcare providers and family members, which not only alleviated despair and helplessness but also helped them rediscover the value of life, reorganize daily routines, and gradually reintegrate into society [24]. As one participant vividly described: “I wanted to give up several times, but all those cousins gave me money and called to tell me to stick with the treatment.” Peer support likewise played an important role, motivating patients to face their illness actively and improving both treatment willingness and adherence [25]. Therefore, by increasing the level of understanding of the disease among family members, they can provide more consistent and stable support and companionship based on a thorough understanding of the disease’s characteristics and the patient’s needs, thereby creating a sense of safety and psychological belonging for the patient. At the same time, patients can access peer support through the psychological support platforms established by the hospital, enhancing their self-management abilities and building supportive relationships through the sharing of experiences and emotional interactions, which in turn boosts their confidence and adaptability in facing the disease.

## Study limitations

The participants in this study were all recruited from a single tertiary hospital, which limits the representativeness of the sample. Therefore, to further validate and refine the research findings, future studies are recommended to adopt a multi-center approach. This will facilitate a more comprehensive and reliable exploration of demoralization syndrome in the context of decompensated cirrhosis.

## Conclusions

This study utilized semi-structured interviews to explore the real-life experiences of demoralization syndrome in 18 patients with decompensated cirrhosis. Through this qualitative approach, four key themes and 11 sub-themes were identified, each focusing on the sources, impacts, coping strategies, and support needs associated with demoralization syndrome. As such, it is imperative for healthcare professionals to take these factors into full consideration when formulating intervention programs, recognize patients’ demoralization syndrome at an early stage, guide them to adopt positive coping strategies, provide avenues for emotional catharsis, and establish an effective social support system, so as to enhance both the physical and mental well-being of these patients.

## Supporting information

S1 File The Mandarin version of the Demoralization Scale. This figure shows the full Mandarin translation of the Demoralization Scale used in this study.
(PDF)

S2 File Semi-structured interview guide example questions. This table provides an overview of the semi-structured interview guide used in our study.
(PDF)

S3 File COREQ checklist. The completed 32-item COREQ checklist submitted to demonstrate adherence to the reporting guidelines.
(PDF)
