# Supplementary material for: The experience of demoralization syndrome in patients with decompensated cirrhosis: A qualitative research
Source: PLoS One. 2025 Dec 1;20(12):e0337182. doi: 10.1371/journal.pone.0337182 (PMC12668536; doi:10.1371/journal.pone.0337182)
Supplement: S1 File — This figure shows the full Mandarin translation of the Demoralization Scale used in this study. (PDF) [file pone.0337182.s001.pdf]

Demoralisation Scale

Name:

Date:

For each statement below, you are asked to indicate how strongly the statement has applied to you over the last two weeks by circling the corresponding number.

Over the past two weeks how often have you felt...

	Never	Seldom	Some-times	Often	All the Time
**1. There is a lot of value in what I can offer others**	0	1	2	3	4
**2. My life seems to be pointless**	0	1	2	3	4
**3. There is no purpose to the activities in my life**	0	1	2	3	4
**4. My role in life has been lost**	0	1	2	3	4
**5. I no longer feel emotionally in control**	0	1	2	3	4
**6. I am in good spirits**	0	1	2	3	4
**7. No one can help me**	0	1	2	3	4
**8. I feel that I cannot help myself**	0	1	2	3	4
**9. I feel hopeless**	0	1	2	3	4
**10. I feel guilty**	0	1	2	3	4
**11. I feel irritable**	0	1	2	3	4
**12. I cope fairly well with life**	0	1	2	3	4
**13. I have a lot of regret about my life**	0	1	2	3	4

**14. Life is no longer worth living**	0	1	2	3	4
**15. I tend to feel hurt easily**	0	1	2	3	4
**16. I am angry about a lot of things**	0	1	2	3	4
**17. I am proud of my accomplishments**	0	1	2	3	4
**18. I feel distressed about what is happening to me**	0	1	2	3	4
**19. I am a worthwhile person**	0	1	2	3	4
**20. I would rather not be alive**	0	1	2	3	4
**21. I feel sad and miserable**	0	1	2	3	4
**22. I feel discouraged about life**	0	1	2	3	4
**23. I feel quite isolated or alone**	0	1	2	3	4
**24. I feel trapped by what is happening to me**	0	1	2	3	4

Scoring instructions: Reverse score asterisked items; then sum item scores
