# Supplementary material for: The experience of demoralization syndrome in patients with decompensated cirrhosis: A qualitative research
Source: PLoS One. 2025 Dec 1;20(12):e0337182. doi: 10.1371/journal.pone.0337182 (PMC12668536; doi:10.1371/journal.pone.0337182)
Supplement: S2 File — This table provides an overview of the semi-structured interview guide used in our study. (PDF) [file pone.0337182.s002.pdf]

Interview Guide

1. Can you share your experiences since your diagnosis?
2. What did you feel most helpless, frustrated, or defeated about when you learned of your diagnosis? What were those feelings like?
3. What do you think these feelings are related to?
4. How did you cope with these psychological reactions?
5. In what ways do you think the people around you (family, friends, or healthcare professionals) could help you? Do you have any other thoughts or feelings?
